# A Scoping Review on Medicinal Properties of *Piper betle* (*Sirih*) Based on Malay Medical Manuscripts and Scientific Literatures

**DOI:** 10.21315/mjms2023.30.5.3

**Published:** 2023-10-30

**Authors:** Nur Fatin Idayu Zamri, Mohd Affendi Mohd Shafri, Zaitunnatakhin Zamli, Suhana Mamat

**Affiliations:** Department of Biomedical Science, Kullliyyah of Allied Health Sciences, International Islamic University Malaysia, Pahang, Malaysia

**Keywords:** Malay medical manuscript, Piper betle, medicinal properties, antimicrobial, analgaesic

## Abstract

**Background:**

Malay medical manuscripts have deciphered the medicinal value of *Piper betle* (*sirih*) enormously. In this review, an effort was made to explore the medicinal use of *P. betle* and correlate this information with the scientific evidence.

**Methods:**

The information regarding the use of *P. betle* was retrieved from the books consisting of a Malay medical manuscript with an identification number MSS 2219 from the National Library of Malaysia. PubMed, ScienceDirect and Scopus databases were used to collect information regarding the scientific evidence for the medicinal use of *P. betle*. This review was written following the Preferred Reporting Items for Systematic Reviews and Meta-Analyses (PRISMA) guidelines. The keywords used for searching the articles included *P. betle*, antimicrobial, analgaesic, haepatic and gastric.

**Results:**

MSS 2219 showed that *P. betle* has varied medicinal uses and based on that, it can be grouped into six categories. *P. betle* application method was different in different conditions. In terms of the literature search, 226 articles were found, 75 articles were extracted for detailed analysis and only 23 met the inclusion criteria. The information was related to the chemical assays, in vivo and in vitro studies.

**Conclusion:**

In summary, *P. betle* has the potential to treat medical conditions in various types of categories as recorded in the Malay medical manuscripts and also based on scientific publications. For clinical purposes, more information is required, such as the specific mechanism involved, the best extraction method and the best dosage for treatment.

## Introduction

Since ancient times, herbs and medicinal plants have been explored for their medicinal value. Their various medicinal properties have been deciphered for use in primary healthcare of both humans and animals. Ancient literature and scholars believed that herbs were the only solution for treating various ailments. Their formulation and practices of herbal medicines have been widely studied and implemented across the globe (1).

Nearly 25% of modern drugs and 60% of antitumour drugs have been derived from natural products (2). In developing countries, around 65% to 89% of people use natural products as remedies for various diseased conditions (2). This widespread use of medicinal plants has attracted researchers worldwide a lot in the last two decades to conduct various studies exploring the pharmacological importance of these plants.

*P. betle* is one of the medicinal plants used in different regions of Asia. This plant has traditional value and is considered auspicious in India, Nepal, Thailand, Vietnam, including Malaysia. Apart from its use in various festivals, ceremonies and rituals, it has been considered beneficial for health.

Malay medicines have been popular for centuries and widely accepted by communities in the Malay Archipelago. The information regarding traditional medicines has been recorded in various sources, both orally and in writing. These written records are known as Malay medical manuscripts, which were handwritten manuscripts in Malay more than 50 years ago. These ancient writings were prepared somewhere in the Southeast Asian region and document the traditional remedies to treat various diseases obtained from local plants, animals and minerals by the people of the Malay civilisation. These manuscripts are an important source and evidence for the local medicines used in the Malay civilisation. These, therefore, serve as a rich source of information regarding the indigenous Malay concept of healing and sickness, medical terminologies, and various physical and non-physical interventions for the present generation.

Most of the herbs were recorded for their medicinal properties, accessibility and ease of use. Noticeably, the description of *P. betle* for the treatment of various diseases was consistently documented by Malay medical practitioners. Most of the books or writings were based on the observations, oral traditions and experiences of shamans or amateurs at that time. Moreover, *P. betle* holds significant importance still in modern medicine due to its vast range of medicinal properties and is in use by the local community for various ailments (3).

Nevertheless, understanding the scientific aspects behind the medicinal properties of any herbs/medicinal plants is of utmost importance for their safer use. Keeping this in view, a scoping review was conducted to explore the use of *P. betle* in Malay medical manuscripts from the scientific aspects. Journal or research articles based on experimental studies were the source of information regarding the medicinal properties of *P. betle*. The focus was driven on the important phytochemicals that may have contributed to the various medicinal properties of *P. betle*.

## Methods

### Data Collection from Malay Medical Manuscript

Transliterated Malay medical manuscripts and publications based on the data extracted from the manuscript are very limited. Due to these limitations, a Malay medical manuscript from the National Library of Malaysia collection coded as Manuscript MSS 2219 was chosen because of its easy access to the manuscript. The following books: *Kitab Tib Harun Mat Piah and Kitab Perubatan Melayu Sari Segala Ubat Tabib Diraja Kesultanan Pontianak* were also taken as the main data sources. The Manuscript MSS 2219 was transliterated. Medical usages of *P. betle* were searched in the manuscript and the books based on the keywords viz. *sirih* or *sirih pinang* or *sirih bertemu urat* and recorded. Since the manuscript was written in the old Malay language, the diseases were recorded based on their local names, such as *demam kepialu angin*. For conducting the scientific discussion, based on our knowledge as native Malay speakers and online search in non-scientific articles, the medicinal properties of *P. betle* were put into several general scientific categories. In short, the medicinal properties of *P. betle*, as mentioned in the manuscripts, were categorised as antimicrobial, analgaesic, hepatoprotective, gastroprotective, respiratory issues and postpartum disorder. Transliteration and data extraction were done by the researcher NFIZ.

### Data Collection from Scientific Findings

**Figure 1:**
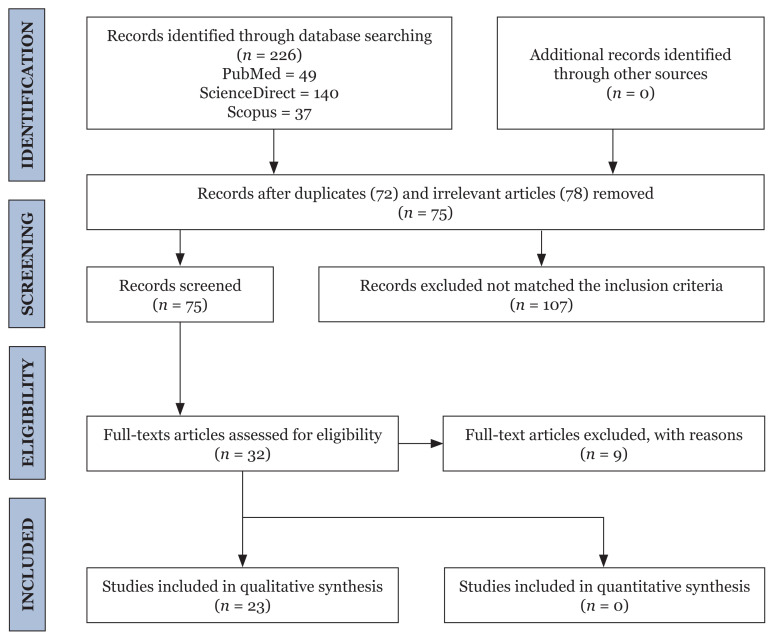
Summary of the search strategy employed according to PRISMA 2009 Flow Guidelines

Various scientific databases were searched for articles related to the medicinal properties of *P. betle* only in four categories mentioned in the manuscript, i.e. antimicrobial, analgaesic, hepatoprotective and gastroprotective. The search engines used in this review were PubMed ( http://www.ncbi.nlm.nih.gov/pubmed/), ScienceDirect (http://www.sciencedirect.com) and Scopus (https://www.scopus.com/). Due to the vast range of medicinal properties *of P. betle* available in the literature, the search keywords used were ‘*Piper betle* AND antimicrobial’, ‘*Piper betle* AND analgaesic’, ‘*Piper betle* AND hepatic’ and ‘*Piper betle* AND gastric’. The data was recorded based on the guidelines of the Preferred Reporting Items for Systematic Reviews and Meta-Analyses (PRISMA) checklist (4). The flow of the PRISMA diagram is shown in Figure 1.

The populations (P), interventions (I), comparators (C), outcomes (O) and study designs of interest (S) or PICOS approach was used to select the relevant articles to be included in this review (4). Based on PICOS, *P. betle* was considered as the population. The interventions involving *P. betle* were not discussed further in this review. The outcome was its various properties, i.e. antibacterial agent, analgaesic, hepatoprotective and gastroprotective properties. Only the original papers of experimental works were selected for this review.

The articles were screened twice to ensure the relevance of the selected articles with the aim of the review. In the first screening, published research articles with a lack of information on the medicinal properties of *P. betle* were excluded. Studies other than experimental work and with no relevance to the present study were also excluded. Therefore, the study titles that mentioned the studies as review, survey, case report and epidemiological study were excluded. This also included titles that were not relevant to this study in the context of medicinal properties, such as anticancer or antidiabetic. All the duplicates among the three databases were removed. The remaining articles which fulfilled the keyword criteria were imported into the Mendeley database. These articles were further evaluated thoroughly for eligibility based on all the inclusion criteria. As mentioned above, the inclusion criteria for the present study were experimental studies conducted in vitro and in vivo and full-text journals published from 1990 to 2020.

## Results

Overall, the terms used for *P. betle* in Malay were identified which included *sirih*, *sirih pinang* and *sirih temu urat* in the manuscript; *Kitab Tib Harun Mat Piah, Kitab Perubatan Melayu Sari Segala Ubat Tabib Diraja Kesultanan Pontianak* and manuscript MSS 2219 from National Library of Malaysia (5–7). Various diseases against which *P. betle* was used and various methods of its use have been mentioned in the manuscripts, which have been summarised in Table 1. The medicinal usage of *P. betle* by the local community in ancient times was categorised into several categories to ease the search for scientific articles.

### Data from Malay Medical Manuscript

Most of the ingredients and all the traditional methods extracted from the manuscript were not translated into English to avoid misinterpretation of the content. Based on our references for Malay medical manuscripts, there were 12 conditions treated with *P. betle*. The conditions included typhoid fever, toothache, yaws, shingles, eye infection, menstrual condition, period pain, liver problem, stomach conditions (stomach upset and vomiting of blood or heartburn), cough, phlegm and postpartum disorder with vomiting. Based on these ailments, the medicinal properties of *P. betle* were categorised into six possible categories. *P. betle* was used differently for the treatment of different diseases. However, all the treatment regimens required a combination of *P. betle* with other ingredient(s). The only treatment of liver-related problems required one more ingredient other than *P. betle*. As for others, two or more ingredients were required. For instance, treatment of typhoid fever requires up to eight other ingredients. A summary of the medicinal use of *P. betle* in Malay medical manuscripts has been summarised in Table 1.

### Data from Scientific Publications

Scientific publications for each medicinal property of *P. betle* mentioned in Malay medical manuscripts were searched. However, only four categories could be aligned with the available scientific literature based on our inclusion criteria, viz. antimicrobial, analgaesic, hepatoprotective and gastroprotective problems. Overall, a search in the databases retrieved 226 articles (PubMed - 49 articles, ScienceDirect - 140 articles and Scopus - 37 articles). Screening of the titles and the abstracts of the identified article revealed 72 duplicates and 78 irrelevant articles. The remaining 75 articles were subjected to full-text assessment based on the selection criteria. Only 23 studies fulfilled the inclusion criteria and provided sufficient data for the qualitative analysis.

Of these 23 studies, a total of 11 studies explored the antimicrobial property of *P. betle* (8–18). Among these, four studies evaluated the antimicrobial properties of *P. betle* alone while the rest of the studies used *P. betle* in combination with other herbs. As for studies on gastroprotective properties, only one out of four studies used *P. betle* alone without other herbs. Furthermore, all the studies which evaluated the analgaesic (19–21) and hepatoprotective (22–26) properties of *P. betle* were specifically conducted using *P. betle* alone. Six studies were based on in vitro studies and chemical assays, while six others relied on in vivo studies and other chemical assays. Five studies were conducted to evaluate the hepatoprotective and gastroprotective properties of *P. betle* which included in vivo, in vitro experiments along with various chemical assays (27–30). Five studies provided in vitro data for the antimicrobial activity of *P. betle* and we found only two studies that explored the analgaesic effect of *P. betle* in vivo.

### Scientific Articles on Antimicrobial Properties

The highest number of publications were found showing the antimicrobial effect of *P. betle* on various types of microorganisms. Most of the selected scientific publications showed the relationship of *P. betle* with the bacteria of the oral cavity and upper respiratory part. *P. betle* was also found to have antibacterial properties against *Streptococcus mutans, Porphyromonas gingivalis* and *Actinomyces viscosus* which usually colonise the mouth (17). Studies conducted by Kawsud et al. (8) and Sivareddy et al. (16) showed antifungal properties of *P. betle*. It was found to be effective against *Candida albicans* which causes oral candidiasis. The antimicrobial and antifungal effects of *P. betle* have been summarised in Table 2.

### Scientific Articles on Analgaesic Properties

Shanthun-Al-Arefin et al. (19) evaluated the in vivo analgaesic property of *P. betle* in Swiss albino mice against gastric pain. Analgaesic activities of *P. betle* have also been demonstrated by writhing response and pain threshold using the hot plate method (20–21). Results of these studies (Table 3) indicated the strong analgaesic potential of *P. betle*.

### Scientific Articles on Hepatoprotective Properties

A total of five articles were found to evaluate the hepatoprotective property of *P*. *betle* (Table 4). Choudhary and Kale (22) conducted studies related to the hepatoprotective potential of *P. betle* in Swiss albino mice and Young et al. (23) conducted these studies in male Wistar rats. In addition, Saravanan et al. (24) and Pushpavalli et al. (25) demonstrated hepatoprotective properties of *P. betle* using albino Wistar rats and female nulliparous Sprague-Dawley rats (26) as in vivo animal models. Parameters assessed in these studies included assay on liver enzymes, alanine transaminase (ALT), aspartate transaminase (AST), and alkaline phosphatase level (ALP) that showed significant decreased and close to normal levels after treatment with *P. betle*. The animal models used for these studies were based on toxicity induced by carbon tetrachloride (CCl_4_), ethanol, D-Galactosamine and methotrexate-induced toxicity (23–26). *P. betle* treatment in these animals also increased the antioxidant levels, as shown in Table 4. Noticeably, two in vitro studies conducted by Young et al. (23) and Pushpavalli et al. (25) reported that *P. betle* treatment did not change the morphology of the liver cells.

### Scientific Articles on Gastroprotective

Bhattacharya et al. (27) and Banerjee et al. (28) showed the gastroprotective effect of *P. betle* in studies done in Sprague-Dawley rats. Furthermore, *P. betle* has also been found to show a gastroprotective effect in Charles-Foster rats and Swiss albino mice (29–30). The animals in all these studies were ulcerated and then treated with *P. betle*. These studies also related the protective effect of *P. betle* with its antioxidant potential, as shown in Table 5. *P. betle* was found to have high phenolic and flavonoid content when extracted using ethanol. Yadav (30) reported that allylpyrocatechol (APC) is also one of the active compounds which showed a protective effect on mucosa and submucosa layer with little exudates after ulceration.

## Discussion

Malay medical manuscripts are archived in many parts of the world, including Malaysia, Indonesia and London. Apart from Malay, the texts have also been found in other languages, such as Javanese and Sudanese (31). The written texts in Malay were in old *Jawi* writing in the old Malay dialect. Transliterated sources were quite limited. Therefore, we had limited sources for Malay medical manuscripts, which narrowed the extent of available data on the medicinal use of *P. betle* in traditional Malay medicine. We could not deny the possibility of other Malay medical manuscripts that indicated the medicinal properties of *P. betle* not included in this review.

Our search explored the use of *P. betle* for treating at least 12 diseases in traditional Malay medicine. Based on these diseases, the medicinal properties of *P. betle* were categorised into six categories. These categories further enabled the search for scientific publications in the selected databases. In Malay medical manuscripts, most of the diseases treated using *P. betle* were the ones caused by microbes and this category showed the antimicrobial potential of *P. betle*. These included typhoid fever, toothache, yaws, shingles and eye infection. Search conduced for scientific articles also revealed that the highest number of scientific articles were published on the antimicrobial properties of *P. betle*. Kawsud et al. (8) and Sivareddy et al. (16) demonstrated the antimicrobial properties of *P. betle*, which included a broad range of microorganisms, including *Candida albicans* and *Streptococcus* sp. Similar findings were reported by Nair and Chanda (15), which showed the potential of *P. betle* against oral candidiasis or toothache. Moreover, *P. betle* also showed antibacterial potential against *Pseudomonas aeruginosa* which caused eye infection (13, 18). Another recent study by Thamaraikani et al. (32) also suggested the antibacterial potential of *P. betle* against *Salmonella sp*., which causes typhoid fever. However, we did not find any scientific publication which investigated the antimicrobial properties of *P. betle* specifically against *Treponema pallidum* and varicella zoster virus, which cause yaw and shingle, respectively. This needs to be explored by the scientific community.

The antimicrobial properties of *P. betle* could be due to the antioxidant potential present in *P. betle* leaves. Out of 23 studies included in this review, 13 articles measured the antioxidant potential of *P. betle*. This antioxidant potential of *P. betle* has been attributed to the presence of various phytochemicals. High flavonoid and phenolic content have been reported in the crude extract of *P. betle* (27). Characterisation of phytochemicals present in *P. betle* showed that it contained compounds such as eugenol, stigmasterol, 3-hexene-ol, heptafluorob-utyrate, ethyl diazoacetate, 4-(2-propenyl)phe-nol, 3-fluoro-2-propynenitrite, tris(trifluoromethyl) phosphine and 4-chromanol. Sharma et al. (17) had shown that *P. betle* contained hydroxychavicol which contributed to its antimicrobial effect.

Bioactive substances derived from *P. betle* could be used as an alternative and safe treatment strategy for antibiotics which could help in inhibiting the growth of bacteria along with reducing the growing issue of antibiotic resistance due to the ruthless use of antibiotics. Many natural products possess antioxidant activity. Many plant-derived compounds, such as polyhydroxylated flavonoids, can be uptaken by bacterial cells because of their specific structure. The antibacterial mechanism of flavonoids could be due to their potential to impair membrane fluidity, alter cytoplasmic membrane fluidity, and inhibit the bacteria cell wall and cell membrane formation (33).

Moreover, flavonoids may also interrupt the synthesis of nucleic acid and inhibit the respiratory metabolism of bacteria. This could lead to impairment of bacteria’s enzyme activities, further leading to cell death and lysis. In addition, exposure of bacteria to the phenolic contents might also decrease the negative charge of the bacterial cell surface and disrupt the transport of solutes. This would result in hyper acidification in the cytoplasm of the bacteria, leading to protein denaturation which further causes membrane damage (33).

Antioxidants have been explored for their protective role against a broad range of diseases such as metabolic syndrome and cancer (34–35). The etiology and development of these diseases are closely related to oxidative stress and inflammatory response. The presence of antioxidants interferes with these processes and thereby shows a protective effect. Vazhappilly et al. (35) reported that the anticancer activity of flavonoids was due to their antioxidant potential, which reduces oxidative-related damages and also triggers the signaling mechanism which activates the tumor suppressor genes. Apart from antimicrobial activities, records from Malay medical manuscripts showed that *P. betle* was used as an analgaesic to treat conditions related to liver diseases, stomach problems, asthma and postpartum disorder. Most of these diseases were related to inflammation. Therefore, it could be stipulated that there could be a similar mode of action by *P. betle* for treating these diseases.

Record of *P. betle* usage for certain medical conditions in Malay medical manuscripts focused mainly on the treatment protocol for using *P. betle*. These treatment protocols mainly focused on using other materials/ingredients along with *P. betle*, for treatment. Traditional used of *P. betle* showed that it was mixed with other herbs or certain compounds in all medical conditions. This led to the assumption that *P. betle* alone might not be sufficient for the treatment of various ailments or that the traditional method used did not release its full medicinal potential.

On the contrary, all the scientific literature based on the categories derived from Malay medical manuscripts showed that most of the experiments involved treatment with *P. betle* extract alone except for one study conducted by Bhinge et al. (10). Most of the scientific experiments conducted did not include standard drugs as a positive control. It is worth noting that a study by Sivareddy et al. (16) to evaluate the antimicrobial activity of several herbs showed that *P. betle* had the highest microbial properties as compared to the other herbs tested in the study. Moreover, one study found that *P. betle* had higher antinociceptive potential in mice as compared to standard drug aspirin (19). These data suggested that *P. betle* alone has the potential to treat various ailments without the need for any adjuvant.

Manuscripts showed that *P. betle* was either directly consumed or boiled in water for treatment purposes. Boiling in water is a very common phytochemical extraction method for using herbs as medicine traditionally. The usage of water as a medium might be due to its availability and non-toxic consumption. Additionally, the water extraction method might be enough to extract the active components in *P. betle* to cure the disease recorded in the manuscripts.

Scientifically, there are various extraction methods used to extract phytochemicals from *P. betle*. Solvents used in these methods include water and various organic solvents such as phenol, ethanol, methanol, butanol and acetone. Different methods and different types of solvents used yielded different types and amounts of phytochemicals. Azahar et al. (36) reported that bioactive compounds extracted from *P. betle* using the water extraction method included steroids, diterpenes, tannin, cardiac glycosides, flavonoids, saponin, phenols, coumarin and alkaloids. Nguyen and Eun (37) reported that the use of methanol for extraction resulted in the highest amount of total phenolic content from many types of leaves, whereas water extraction resulted in the lowest phenolic content. This indicates that medicinal properties such as the antioxidant potential of *P. betle* extract might vary based on the variation of extraction method used.

Active phytochemicals of *P. betle*, such as polyphenols, were shown to have potent medicinal properties such as cytotoxic effects on harmful microorganisms and cancer cells in vitro (38, 39). On the contrary, in vivo studies showed that polyphenols had poor bioavailability and only a small amount was able to reach the target organs. Nevertheless, studies had also shown that a mixture of polyphenols increased their potency which means that their synergistic action could lead to all these scientific findings supporting the usage of *P. betle* as an adjuvant in Malay medical manuscripts.

As per Malay traditional medicine, leaf picking for medicinal purposes should be done according to the specific method to ensure the optimum effectiveness of the herbs. The leaves should be picked before sunrise and for choosing leaves that had exposure to the sun, the criteria are that they should be of medium size and with dark evergreen colour (40). This was quite confusing as sun is the source of light for plants to conduct the process of photosynthesis. Li and Kubota (41) explained this by the fact that the exposure of plants to different types of growth light significantly affects their phenolic content. Not only that, but Kumar (42) described that climatic change also affects the phytochemicals, phenolic content and antioxidant potential of the plants.

**Figure 2:**
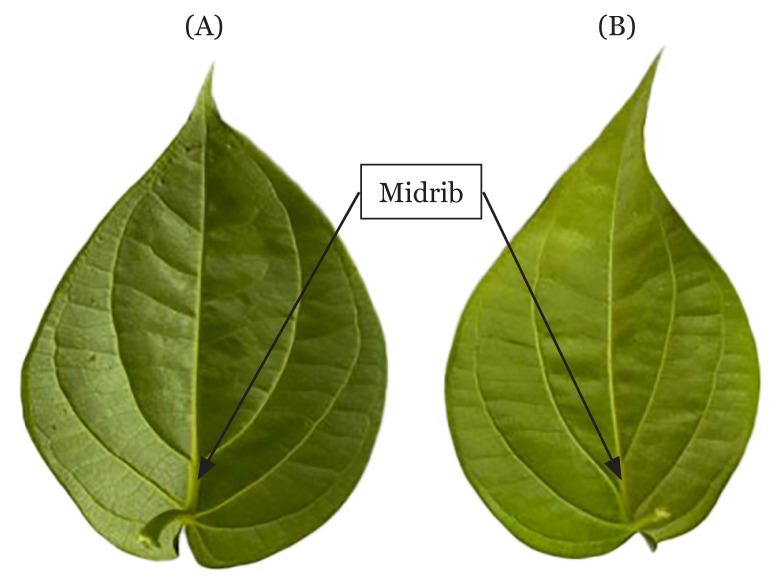
Different pattern of *P. betle*. (A) refer to *‘sirih bertemu urat’* which is completely re-joining veinlet at the midrib of the leaf. (B) refer to *‘sirih tidak bertemu urat’* with unequal re-joining veinlet at the midrib of the leaf

Additionally, for *P. betle*, only the leaves known as *sirih bertemu urat* were believed to have medicinal properties. The *sirih bertemu urat* leaves refers to the leaves where the lateral nerves of the leaf make a complete loop that re-joins with the midrib (Figure 2). The structure of *sirih bertemu urat* is more symmetrical than the common leaves (40). There is no scientific paper yet published that investigated the property of *sirih bertemu urat* or whether the method of leaf picking might affect the yield of active phytochemicals and their potency. This needs to be explored scientifically.

This review was subjected to several limitations. Apart from the limited availability of the transliterated manuscript, the usage of *P. betle* in the manuscript was categorised based on the scientific aspect, instead of the specific diseases in medical terms. The method of *P. betle* applications in the manuscript were also very general in many instances. Therefore, discussion on the scientific aspects such as the mechanism of action involved, dose of the *P. betle* used and duration of treatment was not done in detail. Therefore, this review only provides background data on the significant use of *P. betle* as medicine instead of its conclusive medicinal benefits.

## Conclusion

In summary, current scientific data showed that *P. betle* has potential in the treatment of the diseases recorded in the Malay medical manuscript. But further extensive studies are needed to support this information and the use of *P. betle* in clinics. Available information on the traditional use of *P. betle* as medicine lacks important content, such as the specific number of leaves to be used and frequency of application. Most of the scientific data relate the medicinal properties of *P. betle* with its antioxidant potential. Many active phytochemicals of *P. betle* have also been found to show antioxidant potential. Although many herbs contain a compound with antioxidant properties, different types of compounds have different structures, which might affect their bioavailability, pharmacokinetics and pharmacodynamics. Overall data suggest that future research should focus on the active phytochemicals, dosage, duration and specific mechanisms used by *P. betle* against the pathogenesis of specific diseases. The aspect of the possible usage of *P. betle* as an adjuvant in traditional medicine should also be studied. All this information will provide a strong basis for the use of *P. bettle* as alternative medicine and its commercialisation.

## Figures and Tables

**Table 1:** Usage of *P. betle* as recorded in Malay medical manuscript

Category	Medical indication	Other ingredient	Method according to traditional use	References
Antimicrobial	1. Typhoid fever (*Demam kepialu angin*)	1. *Bakong* leaves, *melada* leaves, *gandarusa* leaves, *malapari* leaves, *munggur* leaves, onions, *Nigella sativa* and *bonglai*	1. Leaves of *bakong* and *sirih pinang* are chewed and then mixed with the other ingredients and spat onto the chest, back and right and left ribs area (*Daun bakong disembur dengan sirih pinang. Seterusnya, semua rempah ini disemburkan dengan sirih pinang pada bahagian tubuh iaitu dada, belakang, rusuk kiri dan rusuk kanan*).	(5)
	2. Toothache (*Sakit gigi*)	2. Roots of *gorak* and roots of *giring*	2. *P. betle* leaves are chewed with the other ingredients (*Sirih bertemu urat dimakan bersama bahan-bahan lain*).	(5)
	3. Yaws (*Penyakit puru*)	3. *Ubat puru parang*, leaves of *orang aring, putor, P. betle*	3. All ingredients are spread on *P. betle* leaves and eaten (*Semua bahan dipalit pada sirih bertemu urat dan makan*).	(5)
	4. Shingles (*Penyakit kayap*)	4. White pepper, galangal (*cekur*), onion, *sirih pinang*	4. *Sirih pinang* and other ingredients are chewed and spat on the diseased area (*Sirih pinang dan semua bahan lain dimamah dan disembur*).	(5)
	5. Eye infection (*Sakit mata*)	5. *Bunga lenga, bunga melur*, white pepper, *lenga*, onion, *kacang hitam, P. betle* (*sirih bertemu urat*)	5. *P. betle* leaves and all ingredients are crushed and mixed with clean water and made into small pellets (*Sirih bertemu urat dan semua bahan dipipis dengan air jernih maka digeliga*).	(6)
Analgaesic	1. Menstrual medicine (*Ubat haid*)	1. *P. betle, gambir*, ginger, *buah terung perat*	1. Half a-bowl of *P. betle* leaves are crushed with other ingredients and the liquid from the mixture is extorted *(Sirih setengah berkas, ditumbuk bersama bahan lain dan diperah)*.	(5)
	2. Period pain (*Ubat senggugut*)	2. Coriander spices *(Rempah-rempah ketumbar), kemukus*, one cup of toasted rice *(secawan beras direndang hangus), P. betle* leaves *(sirih bertemu urat)*, immature betlenut *(pinang muda)*, white pepper	2. All ingredients are crushed until fine, then heated in water just until it cools down, then spat over the person together with *P. betle* leaves (*Rempah rempah dipipis lumat, dijerang pesam-pesam airnya, sirih disemburkan*).	(6)
Hepato-protective	1. Liver problem (*Ubat sakit hati*)	1. Garlic, *P. betle*	1. *P. betle* leaves and garlic are crushed, then hot water is added. Drink or apply as hot compression over the liver (*Daun sirih dan bawang dipipis lumat, tambah air panas, minum atau bungkus dengan daun dan dituamkan pada hati*).	(7)
Gastro-protective	1. Stomach upset with vomiting blood, heartburn (*Senak perut dengan muntah darah, senak ulu hati*)	1. Root of *melada*, root of *maadar*, *P. betle*	1. All ingredients are to be chewed together with *sirih pinang* (*Bahan lain dimamah dengan sirih pinang*).	(5)
Anti-asthmatic	1. Cough medicine (*Ubat batuk*)	1. Root of *terung perat*, pepper, root of *orang aring*, *P. betle*	1. All ingredients are to be chewed together with *P. betle* leaves (*Semua bahan dimakan bersama sirih*).	(5)
	2. To remove phlegm (*Mengeluarkan kahak*)	1. *Sunti karat, buah pelaga, cendana, P. betle (*three yellowed *P. betle leaves*), areca nut, lime	1. Three yellowy leaves of *P. betle* and the other ingredients are crushed finely, wrapped up, steeped in water and drank (*Sirih kuning tiga helai dan semua bahan ditumbuk lumat, bungkus, ambil airnya dan minum*).	(5)
Postpartum Disorder	1. Postpartum disorder with vomiting (*Ubat meroyan muntah*)	1. Onion, *jintan hitam, sirih pinang*	1. *P. betle* and all the ingedients are chewed and spat towards the person *(Semua bahan lain dimakan bersama sirih dan disembur)*.	(5)

Note: *Sirih pinang* = based on Malay context, *sirih pinang* consists of lime and areca nuts wrapped in *P. betle* leaves

**Table 2:** List of studies on antimicrobial activities of *P. betle*

No.	Solvents of extraction	Major phytochemicals profile	Positive control	Results	Summary	References
1.	Ethanol	4-chromanol (62.33%) and eugenol (17.10%) by GC-MS	0.1% CHX (chlorhexidine = antimicrobial therapy for oral candidiasis)	± 0.44 mg/mL and 12.5 ± 0.69 mg/mL of *P. betle* extract removed ≥ 50% and ≥ 90% biofilm formation, respectively. CHX completely inhibit biofilm formation. MICs ranged from 1.56 to 3.13 mg/mL. MFCs ranged from 3.13 to 8.33 mg/mL.	4-chromanol and eugenol might have strong anticandidal activity.	(8)
2.	Ethanol	Heptafluorob-utyrate, Ethyl diazoacetate, 4-(2-Propenyl)phe-nol, 3-Fluoro-2-propynenitrite, eugenol, tris(trifluoromethyl)phosphine) by GC-MS	None	MRSA demonstrated by compounds with *Rf* values of 0.86 and 0.13. Exhibited significant activity against M*β*L-*A*. *baumannii* and CRE-*K*. *pneumoniae*.	Extracts able to deal with medical currently not responsive to existing drugs.	(9)
3.	Mixture of ethanolic extracts of *Azadirachta indica*, *Adhatoda vasica*, *Piper betle*, *Ocinum tenuiflorum and Ponga miapinnata*	None	None	In vitro bacteria study based on formulation of extract mixture (formulation A, B and C). Formulation C showed better zone of inhibition as compared to formulation A, B on *S. aureus, B. subtilis, A. niger* and *E. coli*.	High amount of plant extracts (0.5%) increased the antimicrobial activity of the formulation.	(10)
4.	Ethanol, methanol and supercritical CO_2_ extracts	None	Reference drug controls	MIC for MRSA and VRE strains ranging from 19 μg/mL to 625 μg/mL. MICs for MDR bacteria ranged from 156 μg/mL to 1250 μg/mL. All *P. betle* extracts were bactericidal for all the test MDR bacterial strains in MBC.	PB has the potential to against Gram-positive and Gram negative MDR bacteria.	(11)
5.	Petroleum ether, dichlorometh-ane, chloroform, ethyl acetate and methanol	None	Commercially available alpha-bisabolol purchased from Alfa Aesar	Ethyl acetate extract of P. betle increase prodigiosin inhibitory activity in *S. marcescens* than other extracts. *P. betle* also reduced biofilm thickness.	*P. betle* downregulated the expression of QS regulated virulence genes in *S. marcescens*.	(12)
6.	Ethanol	None	None	*P. betle* reduced biofilm produced by *P. aeruginosa*.	Ethanolic extract of *P. betle* reduce patogenicity of *P. aeruginosa*.	(13)
7.	Hexane, ethyl acetate and ethanol	None	Nystatin as positive control for antifungal. Chlorhexidine as positive controls for antibacterial	Antimicrob properties of *P. betle* was compared with other medicinal plants. Four strains of oral pathogens were tested. *P. betle* showed the strongest antimicrobial (higher than positive control) activity against all tested strains. Extract from ethyl acetate showed strongest antimicrobial activity.	*P. betle* extract using ethyl acetate was the most effective extract against oral pathogenic bacteria and fungi.	(14)
8.	Aqueous and methanol extract	None	Gentamicin, piperacillin, fluconazole	Test on antimicrobial activity of *Terminalia catappa*, *Manilkara zapota* and *P. betle* leaf extract against 10 Gram-positive, 12 Gram-negative bacteria and one fungal strain.	PB was the most active antimicrobial plant.	(15)
9.	Ethanol and ethyl acetate	None	Fluconazole	Antifungal activity of solvent extracts of *P. betle* and *Ocimum sanctum Linn* on *Candida albicans*. *P. betle* extract showed highest inhibition zone, even when compared to positive control. Anticandidal most effective in ethanolic extract of mature leaf and young leaf.	Ethyl acetate extract of *P. betle* was the most effective antifungal against *C. albicans*.	(16)
10.	Methanol	Hydrochavicol	None	In vitro on antimicrobial, antioxidant, and anti-inflammatory activities of extracted Hydroxychavicol from *P. betle*. Hydroxychavicol inhibit growth of *P. gingivalis* and *S. mutans*.	Hydroxychavicol demonstrate antibacterial, antioxidant and anti-inflammatory.	(17)
11.	Methanol	Eugenol, stigmasterol, and 3-hexene-ol	Ampicillin	Eugenol, stigmasterol, 3-hexene-ol were fractinated and tested. Eugonal showed highest antibacterial activity against *E. coli*, *S. aureus* and *P. aeruginosa*. Ampicillin in combination with *P. betle* induced 17%, 60%, 57% and 26% growth inhibition respectively for fractions I, II, III and IV (undisclose fractions) on *P. aeruginosa* and *S. aureus*.	PB fractions target both Gram-negative (*E. coli* and *P. aeruginosa*) and Gram-positive (*S. aureus*) bacteria.	(18)

**Table 3:** Analgesic properties list of studies on analgaesic properties of *P. betle*

No.	Solvents of extraction	Major phytochemicals profile	Positive control	Results	Summary	References
1.	Methanol	None	Aspirin	Study using gastric pain-induced Swiss albino mice. Extract doses of 50 mg, 100 mg, 200 mg and 400 mg per kg body weight reduced the percent in the number of writhings 47.00, 63.28, 69.40 and 71.48, respectively. Aspirin at 200 mg and 400 mg per kg body weight reduce number of writhings to were 51.04 and 67.32, respectively.	The highest dose of the extract showed higher antinociceptive activity than the highest dose of the standard antinociceptive drug tested, aspirin at 400 mg per kg body weight.	(19)
2.	Methanol	None	Nalbuphine	Analgesic activity of *P. betle* extract was evaluated by carageenan-induced paw oedema and hot plate, writhing and formalin tests. *P. betle* extract significanly increase pain threshold and reduced the writhing caused by acetic acid and the number of licks induced caused by carrageenan-induced paw oedema.	*P. betle* extract has strong analgaesic, anti-inflammatory, and antioxidant effects, conforming the traditional use of this plant for inflammatory pain alleviation to its antioxidant potentiality.	(20)
3.	Ethanol	None	Diclofenac	Mice were given acetic acid and number of writhing was recorded. Animals received the drug or extract 1 h prior to administration of acetic acid. The onset of writhing at 25 mg/kg dose is similar with diclofenac. *P. betle* extract in a dose dependent manner significantly reduced acetic acid induced writhing response in mice.	*P. betle* extract has promising analgaesic activity.	(21)

**Table 4:** List of studies on hepatoprotective activities of *P. betle*

No.	Solvents of extraction	Major phytochemicals profile	Positive control	Results	Summary	References
1.	Ethyl alcohol	None	None	In vivo study on Swiss albino mice given different concentration of *P. betle* extract. Hepatic antioxidant status was analysed. *P. betle* inhibited the radiation induced oxidative damage. Endogenous antioxidant also increase in treated groups.	*P. betle* extract inhibited oxidative damage in the liver.	(22)
2.	Water	None	None	Study on carbon tetrachloride (CCl_4_) induced liver injury in male Wistar rat. Rats were treated with extract of *P. betle*. The extract reduced increased level of AST and ALT due to CCl_4_ in dose-dependent manner. The extract also increase SOD and CAT activities.	*P. betle* was able to protect against the liver fibrosis induced by CCl_4_.	(23)
3.	Water	None	None	Study on liver toxicity by inducing ethanol orally to Wistar rats. Assay done on liver enzyme an endogenous antioxidant. Reduced lipid peroxidation and increase endogenous antioxidant following treatment with extract.	*P. betle* exhibits potent antioxidant and hepatoprotective properties in ethanol-treated rat.	(24)
4.	Ethanol	None	None	Study on D-galactosamine-induced hepatotoxic in male rat. Rat treated with either *P. betle*, gum acacia or silymarin. *P. betle* was shown to reduce the high level liver enzymes and increase endogenous antioxidants due to toxicity of ethanol.	*P. betle* leaf extract exhibits potent antioxidant and hepatoprotective properties in hepatotoxic rats.	(25)
5.	Ethanol			Study on adult female nulliparous rats induced with methotrexate-toxicity orally. Treatment with *P. betle* extract showed hepatotoxicity based on liver enzymes and endogenous antioxidant level. Treatment with *P. betle* extract also showed marked reduction in central vein dilatation, absence of leukocyte infiltration, and a reversal to near normal hepatocellular architecture.	PB in a dose-dependent manner attenuated MTX-induced hepatotoxicity, which may be attributed to its ROS scavenging activity, concomitant with an increase in cellular antioxidant activity.	(26)

**Table 5:** List of Studies on Gastroprotective Activities of *P. betle*.

No	Solvents of Extraction	Major Phytochemicals Profile	Positive Control	Results	Summary	References
1.	Ethanol	None	None	In vivo study on indomethacin-induced gastric ulceration of Male Sprague-Dawley rats. Treatment either *P. betle* extract, gum acacia, *E. officinali* or *T. chebula*. P. betle extract showed decreased ulcer index by 78.3, decreased lipid peroxidation by 54.9% and increase DNA by 196%. SOD and CAT were also restored to normal.	The extracts of *P. betle* leaves can act as cytoprotection against gastric lesions, through its antioxidant properties.	(27)
2.	Ethanol	Allylpyrocatechol	None	Study on rats with indomethacin-induced stomach ulceration. Treatment with *P. betle* extract showed reduced ulceration, macroscopically, normal and continuous mucosal layer and formation of the epithelial layer. There were also reduced lipid peroxidation and increased endogenous antioxidant after treatment with extract.	*P. betle* had a potent healing effect on indomethacin-induced gastric lesions in rats.	(28)
3.	Ethanol	None	None	Study on rats induced with ulceration. Treatment was done either with gum acacia or *P. betle* extract. *P. betle* extract was shown to reduce ulcer index, lipid peroxidation and carbonyl content and also increase endogenous antioxidant level.	Antioxidative properties of *P. betle* can act as antiulcerogenic of gastric mucosa.	(29)
4.	Ethanol	Allylpyrocatechol	Omeprazole, misoprostol, trolox and NAC	Study on mice induced with ulceration. Treatment included *P. betle* and 4 positive controls. Histology of mucosa and biochemical markers were measured. *P. betle* treatment showed better results compared to positive controls. Apart from low lesion, it reduced TNF-α gene expression and ROS.	The APC has contributed to its antioxidant action and can treat gastropathy.	(30)
